# Isolation and Characterization of Cat Olfactory Ecto-Mesenchymal Stem Cells

**DOI:** 10.3390/ani12101284

**Published:** 2022-05-17

**Authors:** Marie-Laure Mollichella, Violaine Mechin, Dany Royer, Patrick Pageat, Pietro Asproni

**Affiliations:** 1 Tissue Biology and Chemical Communication Department, Institute of Research in Semiochemistry and Applied Ethology, 84400 Apt, France; v.mechin@group-irsea.com (V.M.); p.asproni@group-irsea.com (P.A.); 2 Independent Researcher, 84440 Robion, France; dany.royer.drv@gmail.com; 3 Research and Education Board, Institute of Research in Semiochemistry and Applied Ethology, 84400 Apt, France; p.pageat@group-irsea.com

**Keywords:** olfactory stem cells, isolation, stemness, differentiation, characterization, regenerative medicine

## Abstract

**Simple Summary:**

Cat’s health is impacted by several diseases and lesions for which cell therapy could be an interesting treatment. Mesenchymal stem cells or adult stem cells are found in developed tissue. Olfactory mucosa contains stem cells called olfactory ecto-mesenchymal stem cells which have already been isolated from various animals as dogs and horses. The aim of this study was to evaluate the feasibility of collecting olfactory ecto-mesenchymal stem cells in cats. For that purpose, four cats were biopsied; the cells were collected and characterized. They show stemness features and differentiation capabilities as all the other mammals previously studied. Therefore, olfactory ecto-mesenchymal stem cells could be a promising tool for feline regenerative medicine.

**Abstract:**

The olfactory mucosa contains olfactory ecto-mesenchymal stem cells (OE-MSCs) which show stemness features, multipotency capabilities, and have a therapeutic potential. The OE-MSCs have already been collected and isolated from various mammals. The aim of this study was to evaluate the feasibility of collecting, purifying and amplifying OE-MSCs from the cat nasal cavity. Four cats were included in the study. Biopsies of olfactory mucosa were performed on anesthetized animals. Then, the olfactory OE-MSCs were isolated, and their stemness features as well as their mesodermal differentiation capabilities were characterized. Olfactory mucosa biopsies were successfully performed in all subjects. From these biopsies, cellular populations were rapidly generated, presenting various stemness features, such as a fibroblast-like morphology, nestin and MAP2 expression, and sphere and colony formation. These cells could differentiate into neural and mesodermal lineages. This report shows for the first time that the isolation of OE-MSCs from cat olfactory mucosa is possible. These cells showed stemness features and multilineage differentiation capabilities, indicating they may be a promising tool for autologous grafts and feline regenerative medicine.

## 1. Introduction

Mesenchymal stem cells (MSCs) are plastic-adherent cells that show self-renewal and high proliferative capabilities and can differentiate into the mesodermal lineage under standard in vitro differentiating conditions [1,2]. Due to these abilities, MSCs have been described as having therapeutic potential in several diseases, such as cancer [3], traumatic brain injury [4], chondral defects [5] or cardiovascular diseases [6], even if these statements should be confirmed by more extensive investigations.

Stem cell-based regenerative medicine is used in veterinary medicine to repair damaged tissue by a disease or injury. Even if this kind of therapy still needs to be more extensively investigated, in the future stem cells may be an alternative treatment in some cases for which the conventional medicines cannot repair the damages tissues. MSCs have differentiation potential but also immune regulatory properties, influence on vascularization, apoptosis, fibrosis and inflammation [7,8,9]. Regenerative cell therapy is used in degenerative diseases (heart failure), immune mediated diseases (feline asthma, canine atopic dermatitis, feline chronic gingivostomatitis) [10], inflammatory diseases (wound healing defect). MSCs induce immune enhancing response and have an anti-inflammatory effect [10].

In cats, MSCs have been first isolated in 2002 from bone marrow [11], then from fat, fetal fluid, peripheral blood and amniotic membranes [12,13]. Feline MSCs have been already used in trial against gingivostomatitis, enteropathies, chronic kidney disease, asthma, feline eosinophilic keratitis, neurological ailments, cardiomyopathy. These studies gave variable therapeutics results. Concerning the gingivostomatitis, grafts with autologous or allogenic adipose stem cells led to complete remission in 3/7 and 2/7 cats, respectively, with 2/7 cats that presented a substantial improvement in both cases [14,15]. In acute and chronic asthma, Trzil and colleagues showed that the graft of allogenic adipose stem cells can induce an improvement in different parameters, among which airway eosinophilia [16,17]. Good results were also shown in chronic enteropathy treatment with allogenic stem cells administration, with an improvement of clinical signs in 5 of the 7 treated cats [18]. Finally, concerning chronic and acute kidney disease treatment, the results seem less promising, going from a mild decrease in serum creatinine [19,20,21] to no improvement in acute or chronic disease [22,23]. This heterogeneity suggests that more studies are needed to optimize the MSCs administration and sources [12,13].

Among the various sources of MSCs, the olfactory mucosa is a promising candidate for both humans and animals [24,25]. Indeed, the olfactory mucosa contains olfactory ecto-mesenchymal stem cells (OE-MSCs) that are easily accessible and collectable due to their localization [26]. OE-MSCs also show stemness and multipotency capabilities, resulting in therapeutic potential, which has already been demonstrated in several diseases, such as hearing loss [27], cerebral ischemia [28] and Parkinson’s disease [29].

We previously showed that it is possible to collect and isolate olfactory stem cells from various mammals [26], but the feasibility has never been evaluated in cats. Since cats may suffer from several diseases and lesions impacting their welfare [12], stem cells could also be an interesting tool for cell therapies in this species, and OE-MSCs identified in the olfactory mucosa are promising candidates for autologous grafts [24].

The aim of this study was to investigate the feasibility of isolating OE-MSCs from cats and characterizing these cells.

## 2. Materials and Methods

### 2.1. Ethics Statement

This study was conceived and performed in accordance with French (2013-118) and European law (2010/63/EU) on the protection of animals used for scientific purposes. This protocol was approved by the Ministry of Higher Education, Research and Innovation of France and by the IRSEA’s Ethics committee C2EA125 (approval number: UE-2018-EU0552).

### 2.2. Biopsy of Olfactory Mucosa and Isolation and Expansion of OE-MSCs

Four healthy cats from IRSEA’s facilities were included in the study (2 males, 2 females; 6 ± 4.6 years). Since the cats belonged to our facilities, the health status was daily monitored by our veterinary team. Sedation was performed with ketamine (Imalgene 1000, Merial SAS, Lyon, France) (10–20 mg/kg, sc), medetomidine (Domitor, vetoquinol, Lure, France) (50 µg/kg, sc) and butorphanol (Dolorex, MSD Santé Animale, Beaucouzé, France) (0.4 mg/kg, sc). Then, anesthesia was induced with propofol (Propovet, Zoetis, Malakoff, France) (1.2 mg/kg) and maintained with isoflurane (Belamont) (2%). The biopsies of olfactory mucosa were performed by nasal cavity exploration with a common rigid biopsy forceps on anesthetized animals. To reach the olfactory mucosa, as for the other domestic species, the forceps were inserted into the nasal cavity until its caudal limit. For each cat, 2 biopsy samples (1 per side) were obtained and placed at 4 °C in culture medium Dulbecco’s Modified Eagle’s Medium/Ham’s F12 (DMEM/F12, 1% GlutaMAX, Pan Biotech, Aidenbach, Germany, cod. P04-41150) supplemented with 10% serum (fetal bovine serum, (FBS, Dutscher, Bernolsheim, France), 2% penicillin and streptomycin 100X (P/S) (Dutscher, Bernolsheim, France, cod. L0022-100), and 2.5 mg/mL amphotericin B (Hyclone, Marlborough, MA, USA, cod. SV30078.01) until culturing. The olfactory mucosa biopsies were washed in DMEM/F12 medium and were mechanically dissociated using 25-gauge needles to obtain pieces of a few square millimeters. Each pieces of biopsy were placed in a 2 cm^2^ culture well coated with poly-L-lysine (PLL, Sigma-Aldrich, Saint-Louis, MO, USA cod. P1274) with 200 µL of the culture medium described above for 1 week. When the explants adhered to the plate, the wells were filled with 400 µL of culture medium. Two weeks after plating, the concentration of antibiotic and amphotericin B were halved. The medium was renewed every two to three days. When confluence was reached, the cells were detached, dissociated with trypsin EDTA solution (0.25%, Dutscher, Bernolsheim, France, cod. L0931-100), pooled, centrifuged at 300× *g* for 5 min and replated at lower density.

### 2.3. Generation of Spheres

Cells were counted on Kova slides (Dutscher, Bernolsheim, France, cod. 050126) and, plated at a density of 30,000 cells/cm^2^ in PLL-coated dishes (5 μg/cm^2^) and fed with serum-free DMEM/F12 culture medium supplemented with 1% P/S, 1% insulin, transferrin, selenium (ITS-X, Gibco, cod. 51300044), 50 ng/mL epidermal growth factor (EGF, Gibco) and 50 ng/mL fibroblast growth factor 2 (FGF, Gibco, cod. PHG0311L). This culture medium was renewed every two days. After one week of treatment, spheres were observed with an inverted microscope.

### 2.4. In Vitro Neural Lineage Differentiation Assays

For neuronal differentiation, OE-MSCs after sphere generation were grown under two culture conditions as described previously [30,31]. The spheres were dissociated with trypsin EDTA solution and plated at a density of 15,000 cells per cm^2^ in a culture well (2 cm^2^) on glass coverslip coated with PLL. The cells were cultured in two different media: DMEM/F12 Glutamax, 1% P/S, 1% FBS, 2% B-27 Supplement (Gibco, cod. 17504044), 1% N-2 Supplement (Gibco, cod. 17502048), 10 ng/mL EGF, 20 ng/mL FGF or DMEM/F12 Glutamax, 1% P/S, 1% FBS, 2% B-27 Supplement, 1 mM Valproic acid (Sigma-Aldrich, Saint-Louis, MO, USA, cod. P4543). The medium was renewed every two days for one week. For confirmation of the differentiation, the cells were fixed in paraformaldehyde solution (4%, Alfa Aesar, Haverhill, MA, USA, cod. J61984), and immunocytochemistry (ICC) of the Glial fibrillary acidic protein (GFAP) and Microtubule Associated Protein 2 (MAP2) proteins was performed as described in Section 2.9 with the antibodies in Table 1.

### 2.5. Expression of Nestin

OE-MSCs (passage 6) were plated on glass coverslips in a 24-well plate at a density of 15,000 cells per cm^2^ in growth medium (DMEM, 10% FCS, 1% P/S, 1.25 mg/mL amphotericin B) for approximately 48 h. The cells were then fixed in a paraformaldehyde solution (4%) and ICC was performed as described in Section 2.9 with the antibodies that are reported in Table 1.

### 2.6. Clonal Efficiency Assay

OE-MSCs (passage 7) were plated in 6-well plates at a density ranging from 10 to 320 cells/well in triplicate. After plating, the dishes were placed at 37 °C, in a humidified, 5% CO2 atmosphere for 7 days. The culture medium (DMEM, 10% FCS, 1% P/S, 1.25 mg/mL amphotericin B) was renewed every two days. The colonies were paraformaldehyde-fixed during 15 min at room temperature (RT). Colonies were stained for 30 min using crystal violet, rinsed with tap water bath and let dry at RT. Then, the colonies were observed with an inverted microscope and manually counted. For each sample, clonal efficiency (% of clonogenicity) was calculated as follows:

(mean number of colonies/total number of seeded cells) × 100

When too many colonies overlapped, counting was not performed.

### 2.7. In Vitro Proliferation Assay

The assay was performed on OE-MSCs 2 months (10 passages) and 3 months (20 passages) after the initial plating. The cells were seeded in 96-well plates in triplicate and counted with CellTiter 96 Aqueous One Solution Reagent (Promega, Madison, WI, USA, cod. G3580) according to the manufacturer’s protocol at 8 h, 24 h, 48 h, 72 h, and 96 h after seeding. Briefly, 20 µL of CellTiter was added for 100 µL of culture medium. The plate was incubated at 37 °C in a humidified, 5% CO2 atmosphere for 1 h to 4 h. The absorbance at 490 nm was recorded with a plate reader. The population doubling time (PDT) was calculated as follows:

Duration × ln(2)/ln(FinalConcentration) − ln(InitalConcentration)

### 2.8. In Vitro Mesodermal Differentiation Assays

For osteogenic differentiation, OE-MSCs (passage 8) were grown in DMEM/F12 Glutamax, 10% FBS, 0.1 µM dexamethasone (Sigma-Aldrich, Saint-Louis, MO, USA), 0.15 mM l-ascorbic acid (Sigma-Aldrich, Saint-Louis, MO, USA cod. A92902), and 1 mM Sodium Phosphate Monobasic (Sigma-Aldrich, Saint-Louis, MO, USA) for 21 days. The culture medium was renewed every two days. For analysis of osteogenic differentiation, cell cultures were fixed in a paraformaldehyde solution (4%) for 15 min and stained with von Kossa (Bio-Optica, Milano Italy, cod. 04-170801) or Alizarin Red stain (ScienCell, Carlsbad, CA, USA cod. 8678) according to the manufacturer’s instructions. For chondrogenic differentiation, the cells were grown in pellets in 15 mL polypropylene tube in DMEM/F12 Glutamax, 1% P/S, 0.1 µM dexamethasone, 0.15 mM l-ascorbic acid, 0.35 mM proline (Sigma-Aldrich, Saint-Louis, MO, USA), 1 mM sodium pyruvate (Sigma-Aldrich, Saint-Louis, MO, USA, cod. S8636), 1% ITS, and 10 ng/mL transforming growth factor beta-3 (TGF-β3, Invitrogen, cod. RP-8600) for 21 days and fixed in 10% buffered formalin (pH 7.4), routinely processed and paraffin embedded. Four-micrometer-thick sections were cut and stained with Alcian blue/PAS (Bio-Optica, Milano Italy, cod. 04-163802) according to the manufacturer’s instructions.

For tenogenic differentiation, 30,000 OE-MSCs were grown in 24-well plates on a 5 μg/cm^2^ collagen-I matrix (Gibco, cod. A1064401) in DMEM/F12 Glutamax without FBS, 50 ng/mL Growth Differentiation Factor 5 (GDF-5, R&D Systems, Minneapolis, MN, USA, cod. 8340-G5-050), 50 ng/mL Growth Differentiation Factor 5 (GDF-7, R&D Systems, Minneapolis, MN, USA, cod. 8386-G7-050) and 20 ng/mL TGF-B3 (Invitrogen, cod. RP-8600) for 7 days. The culture medium was renewed every two to three days. For evaluation of tenogenic differentiation, the cells were paraformaldehyde fixed, and ICC was performed against the tenomodulin and scleraxis proteins.

### 2.9. Immunocytochemistry

Immunocytochemistry was carried out to assess the expression of nestin, the neural proteins GFAP and MAP2, and the tenoblast proteins tenomoduline and scleraxis, with the appropriate primary antibody (Table 1).

Paraformaldehyde fixed cells were incubated for 1 h at RT with blocking solution (3% bovine serum albumin (BSA, Sigma Aldrich Saint-Louis, MO, USA, cod. A7030), and 0.1% Triton X-100, (Sigma-Aldrich, Saint-Louis, MO, USA, cod. T8787), 5% goat serum (Dutscher, Bernolsheim, France) in phosphate-buffered saline (PBS, Hyclone, Marlborough, MA, USA, cod. SH30264.01) solution. Glass coverslips were then incubated over-night at RT with the appropriate primary antibody diluted in the staining solution PBS 3% BSA, 5% goat serum). The cells were then rinsed 3 times in PBS and incubated for 3 h with the appropriate AlexaFluor 488-conjugated polyclonal secondary antibody. After several washes in PBS, cells were counterstained with 0.5 μg/mL Hoechst blue (33,258, Sigma-Aldrich, Saint-Louis, MO, USA) for 10 min and mounted with anti-fading medium (ProLong Diamond, Invitrogen, cod. P36965). Negative control conditions were carried out by omitting the primary antibody.

### 2.10. Image Acquisition

Pictures were acquired with an inverted microscope EVOS^®®^ FL Auto Imaging System (Thermofisher, Waltham, MA, USA) and negative controls were used to adjust image acquisition parameters. ICC pictures were acquired with monochrome camera on DAPI (357/447 nm) fluorescence channel for Hoechst staining and GFP (470/525 nm) fluorescence channel for Alexa 488 staining.

Non fluorescent images were acquired with color brightfield image mode.

## 3. Results

### 3.1. Biopsy of Olfactory Mucosa and Isolation and Expansion of OE-MSCs

**Figure 1:**
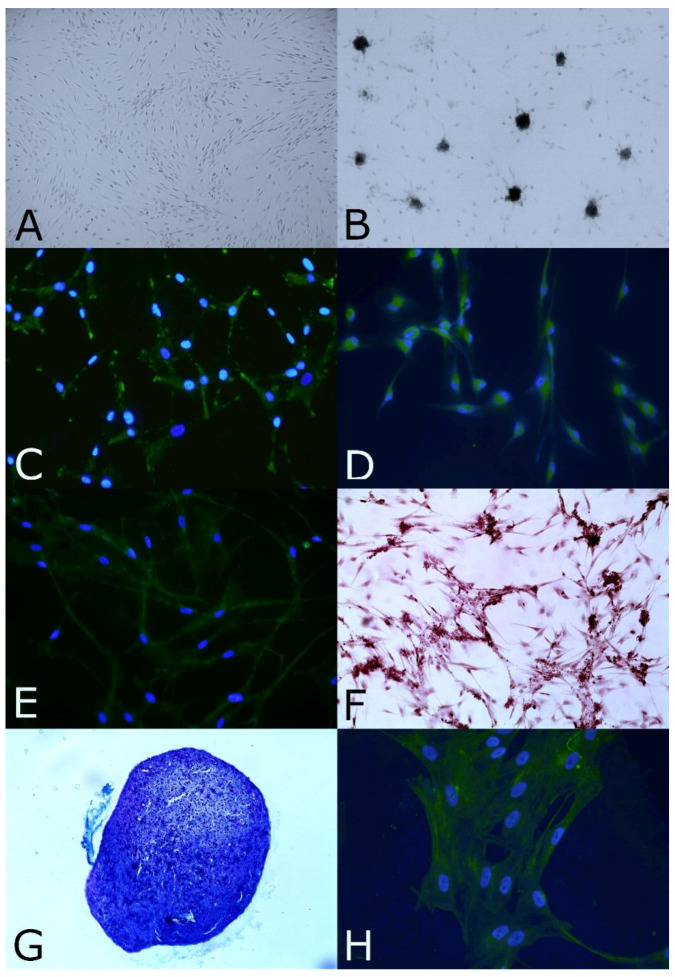
Morphology, stemness features, and assessment of neural and mesodermal differentiation abilities of OE-MSCs in vitro. (**A**) In growth culture medium, the OE-MSCs formed adherent cells with fibroblastic morphology (ob. × 40). (**B**) Grown in specific culture conditions, the OE-MSCs could generate spheres (ob. × 100). (**C**) Cells expressed the nestin protein (in green, ob. × 200). Neural lineage differentiation was assessed with ICC against GFAP (**D**) and MAP2 (**E**) (in green, ob. × 200). (**F**) Osteogenic differentiation was assessed with Alizarin Red, and calcium deposits were positively labeled in brown (ob. × 100). (**G**) OE-MSCs in chondrogenic differentiation medium were positively labeled with Alcian blue/PAS staining (in purple-blue, ob. × 40). (**H**) Tenogenic markers were assessed with ICC against tenomodulin (in green, ob. ×200). For ICC, cells were colabeled with Hoechst (blue).

Olfactory mucosa biopsies were successfully obtained from the 4 anesthetized cats. The only undesirable effect observed immediately after the biopsies was nasal bleeding that was rapidly stopped by applying a sterile gauze upon the nostrils. The animals recovered from anesthesia with no other unwanted side effects. One to two weeks after the biopsies, we observed adherent cells with fibroblastic morphology growing from the explants and forming a homogenous monolayer (Figure 1A).

### 3.2. Stemness and Immature Features

The OE-MSCs displayed nestin protein expression, and under specific culture conditions, these cells could generate spheres, as shown in Figure 1B,C.

### 3.3. Clonal Efficiency Assay

The OE-MSCs formed colonies at a low cell density (20 to 320 cells/well). The average clonal efficiency for the feline OE-MSCs was 10.64% ± 10.48% (mean ± SD).

### 3.4. In Vitro Proliferation Assay

The population doubling times of the feline olfactory stem cells were examined at 2 months (P10: 50.07 h ± 43.13 h (mean ± SD)) and 3 months (P20: 80.69 h ± 22.42 h (mean ± SD)) after the biopsies. The population doubling time increased from P10 to P20

### 3.5. In Vitro Neural and Mesodermal Differentiation Assays

Before neural lineage differentiation, the GFAP and MAP2 proteins were expressed in cells in the basal state (Figure S1A,B in Supplementary Materials). Within in vitro differentiation conditions, ICC demonstrated that the expression of these proteins was increased (Figure 1D,E). Under the appropriate culture conditions, cells expressed biochemical features specific to osteoblasts, chondroblasts and tenoblasts. The differentiated cultures showed Alizarin Red (Figure 1F) and von Kossa (photo not shown) staining after osteogenic differentiation. In chondrogenic differentiation culture conditions, the cells aggregated, and their histological sections were positive for Alcian blue/PAS staining (Figure 1G). The OE-MSCs expressed the tenomodulin protein under tenogenic differentiation conditions (Figure 1H). Consequently, feline OE-MSCs could differentiate into the mesodermal lineage.

## 4. Discussion

Our study showed for the first time that OE-MSCs can be extracted from cat olfactory mucosa. This tissue was easily accessible in the nasal cavity of anesthetized animals, and the sampling presented few technical issues [26]. The Feline OE-MSCs presented fibroblastic-like morphology and two stemness and immaturity features previously described in human OE-MSCs [24], such as nestin protein expression, and the ability to form spheres, even if they were smaller than those in dogs, horses and rabbits [26]. The feline OE-MSCs formed colonies at a low density, which is a characteristic of stem cells. The number of colonies is less high than in the eight mammalian genera already characterized [26]. Clonal efficiency assay or colony-forming unit fibroblast (CFU-f) is used to quantify the number of MSCs progenitors in bone marrow samples. Feline OE-MSCs have better clonal efficiency than MSCs from bone marrow [32] but inferior compared to adipose-derived stem cells [33]. These cells also showed differentiation into neural and mesodermal lineages under appropriate specific culture conditions.

The feline OE-MSCs expanded and amplified rapidly, even if they proliferated slower at P20 than P10 which reveals a decrease of the self-renewal capacity of OE-MSCs. The feline OE-MSCs still have high proliferative capabilities in the early passages even if the PDT is lower than that of dogs, rabbits and horses due to species specific diversity [26]. Moreover, the proliferation rate was the same [33] or better [34,35,36] than that of feline MSCs from other tissues at P10. The feline MSCs seems to have short proliferation capabilities with time and suffer of early senescence [10]. Indeed, feline adipose tissue derived MSCs show a significant increase of the PDT after 4–5 passage [34,35]. Cat peripheral blood MSCs stop proliferate at passage between 7 and 9 [36]. While these MSCs were evaluated only until P10, OE-MSCs showed to be able to proliferate also at P20, suggesting that they possess a longer duration in tested culture conditions. However, in previous studies, feline MSCs have been transplanted much earlier than passage 20, commonly between P2 and P5 [14,17]. In our study we did not evaluate the karyotype of OE-MSCs at P20, thus further analyses should aim to assess if OE-MSCs possess any kind of alteration that make these cells unsuitable for graft at this point.

Similar to those of rats, rabbits, dogs and horses, the feline OE-MSCs expressed GFAP and MAP2 in the basal state, which is a known stemness feature [26]. The expression of these proteins increased after in vitro differentiation (Figure S1A,B in Supplementary Materials), indicating that these cells could differentiate into neural lineages. This finding may open the way for further studies aiming to evaluate if the feline OE-MSCs could represent a potential treatment for brain or neural lesions [28,37].

Our analyses showed that OE-MSCs could also be induced in osteoblast-like, chondroblast-like and tenoblast-like cells under the appropriate differentiation conditions. Even if these are only in vitro findings, they may suggest that future studies could investigate if OE-MSCs may also have a potential role in the treatment of bones, cartilage and tendon lesions. MSCs demonstrated their efficacy in equine tendinopathy [38,39]. They have also shown benefic effect in bone healing in canine, ovine and caprine clinical model [40]. Canine MSCs have cartilage regenerative effect in dog with osteoarthritis [41].

Compared to bone marrow stem cells, OE-MSCs are easier to collect. Indeed, olfactory mucosa biopsy could be performed during a routine intervention requiring simple anesthesia. Since the olfactory mucosa is easily accessible, sampling is safer and less painful than bone marrow biopsy. On the other hand, the adipose stem cells are the most largely studied in cats, since they are easy to collect and possess a high proliferative ability [12], even if this capability seems to be reduced after 4–5 passages [34,35]. However, the aim of this study was to explore and propose another source of feline MSCs and to expand the knowledge on feline stem cells.

## 5. Conclusions

This study showed for the first time that the olfactory mucosa is a source of MSCs in cats. These cells can be easily isolated and amplified. Feline OE-MSCs display stemness characteristics and differentiation capabilities. These results pave the way for further studies that should evaluate if OE-MSCs could be a promising tool for feline autologous stem cell therapy and for veterinary regenerative medicine.

## Figures and Tables

**Table 1:** Antibodies used for immunocytochemistry.

Antibody	Target	Host	Supplier	Reference	Dilution	Secondary Antibody
Anti-nestin	Stemness marker	Rabbit	Abcam	ab7659	1/500	Alexa Fluor 488
Anti-GFAP	Neural marker	Chicken	Abcam	ab4674	1/500	Alexa Fluor 488
Anti-MAP2	Neural marker	Chicken	Abcam	ab5392	1/500	Alexa Fluor 488
Anti-tenomodulin	Tenoblast marker	Rabbit	Abcam	ab81328	1/250	Alexa Fluor 488
Anti-scleraxis	Tenoblast marker	Rabbit	Abcam	ab58655	1/250	Alexa Fluor 488

## Data Availability

The data presented in this study are available in the article and in the Supplementary Materials (Figure S1).

## Supplementary Materials

The following supporting information can be downloaded at: https://www.mdpi.com/article/10.3390/ani12101284/s1, Figure S1: Negative controls of histochemical and immucytochemical analyses.
