# Supplementary material for: Isolation and Characterization of Cat Olfactory Ecto-Mesenchymal Stem Cells
Source: Animals (Basel). 2022 May 17;12(10):1284. doi: 10.3390/ani12101284 (PMC9137790; doi:10.3390/ani12101284)
Supplement: Supplementary file 1 [file animals-12-01284-s001.zip › animals-1671247-supplementary.pdf]

animals

MDPI

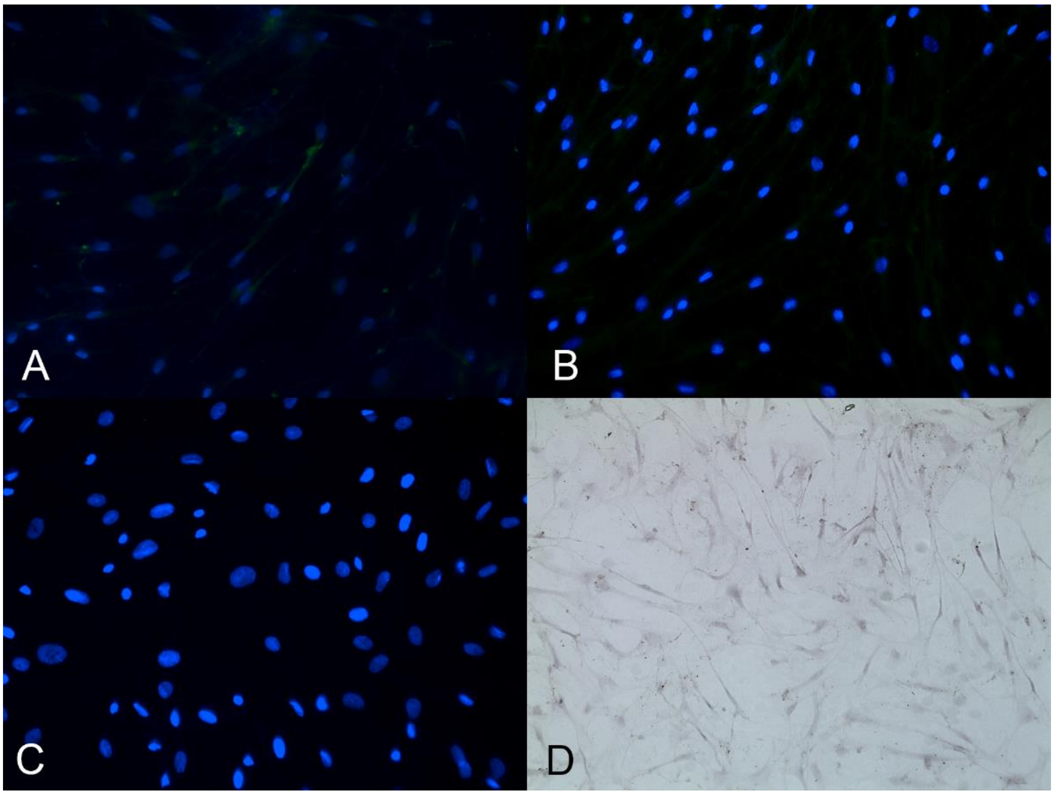

Figure S1. Negative controls of histochemical and immucytochemical analyses. Before neural differentiation, feline OE-MSC showed a basal weak positivity against GFAP (**a**) and MAP2 (**b**) (in green, ob. x 200). (**c**) No immunoreactivity was observed against tenomodulin (ob. x 200). (**d**) No positivity was observed in negative controls for Alizarin Red stain (ob. x 100).
